# Experience and local memory of older people in the face of disasters: a systematic review

**DOI:** 10.3389/fpubh.2023.1163561

**Published:** 2023-05-24

**Authors:** Camila Navarrete-Valladares, José Sandoval-Díaz, Eduardo Sandoval-Obando

**Affiliations:** ^1^Doctorado en Psicología, Universidad de Concepción, Concepción, Chile; ^2^Centro de Estudios Ñuble, Universidad del Bío-Bío, Chillán, Chile y Centro Interuniversitario de Envejecimiento Saludable (CIES), Chillán, Chile; ^3^Claustro Doctorado en Ciencias Sociales, Universidad Autónoma de Chile, Temuco, Chile

**Keywords:** older people, climate change, disasters, vital experience, collective memory

## Abstract

**Introduction:**

The increase in population aging establishes new risk scenarios in the face of the intensification of disasters due to climate change; however, previous experiences and collective memory would generate opportunities for older people to acquire adaptive and coping capacities in the face of these events.

**Objective:**

To analyze the theoretical-methodological characteristics presented by the studies carried out between the years 2012 and 2022 about the experience and collective memory of the older adult in the face of climate change.

**Method:**

A systematic literature review (SLR) was carried out following the guidelines of the PRISMA statement. The databases consulted were Web of Science, Scopus, EBSCO host, and Redalyc, selecting 40 articles in Spanish, English, and Portuguese.

**Results:**

The importance of experience and collective memory in the face of disasters as an adaptive factor in older people was identified. In addition, sharing experiences allows them to give new meaning to what happened, emphasizing confidence in their personal resources and self-management capacity and fostering perceived empowerment.

**Discussion:**

It is essential that in future studies the knowledge provided by the older adult can be privileged, recognizing the importance of their life histories and favoring the active role in their development and wellbeing.

## Introduction

Climate change has become one of the main risks that increase vulnerability to natural disasters, so it is essential to provide opportunities for the most exposed groups to acquire adaptation and coping capacities to face this problem on a global and local scale (1).

In this context, it is necessary to strengthen those societal and community measures that foster adaptive capacity and reduce vulnerability to disaster risk processes intensified by climate change, especially in the most susceptible groups, such as the older adult population (2, 3).

In addition to this scenario of global environmental crisis, there are statistical projections on the accelerated population aging (4), under which one out of every 11 older adult people living in underdeveloped countries is exposed to climate risks (5, 6).

In terms of the susceptibility of the older adult population to climate change, it is important to consider various aspects, such as mobility difficulties in evacuation and emergency processes, as well as the morbidities inherent to the evolutionary stage in which they find themselves (7–9).

On the other hand, the literature highlights that a population that manages to adapt to climatological stressors is considered less vulnerable to the impacts of climate change, due to the deployment of coping strategies that can absorb, recover, and/or resignify the potentially traumatic event in a resilient manner (10–13).

Among the studies that address the coping strategies used by the older adult population in the face of disaster risk processes, the importance of access to communication and dissemination technologies (14), recognition of safe zones within the home (15), and early warning systems (16) are noted. Likewise, in collective terms, collective memory has been recognized as a central community strategy because of the possibilities of resignification that it gives to the lived experience, as well as the intersubjective understanding of the stages of the disaster that occurred (17, 18), enabling the maintenance of a high collective awareness of the risks (19).

Another relevant individual capacity is life experiences in risk situations, which determine to a large extent the presence of those affected by disasters, which is conditioned by multiple factors such as identity, personality type, lifestyle, and living conditions, to mention just a few (20, 21). Within this field, there is research that indicates that sharing a strongly shocking collective experience tends to increase cohesion, operating as an instance of support, containment, and post-disaster repair (22, 23).

Therefore, and in line with the importance of both capacities to adapt to climate change in the older population, we argue the need to deepen the existing scientific evidence, on the experience and collective memory in the face of climate change (11, 24). To accomplish the above, we conducted a systematic literature review (SLR) of empirical research published between the years 2012 and 2022, that addressed the relationship between previous disaster experiences and/or collective memory of older people in the face of climate change risks. For this, we set the following objectives: (i) to identify authors, countries of the studies, types of memory and/or experiences, types of risk and/or disaster of natural origin associated, and sources of information and methodology used; (ii) to analyze the relationship of previous experiences of disaster and/or collective memory of the older adult to the risks of climate change; and (iii) to identify the lessons learned from the experiences and/or collective memory of the older adult population in the face of the risks of climate change.

## Methods

A systematic review of the literature (SLR) was carried out following the guidelines and recommendations of the Preferred Reporting Items for Systematic Reviews and Meta-Analyses (PRISMA) statement, complying with points 1–10, 13, 16–17, 20, and 23–27, from their checklist (25). In turn, following the PICo format of qualitative research, we pose the following research question: What role do previous disaster experiences and the collective memory of older people play in the face of climate change risks?

### Search strategies

The search for articles was limited to studies with empirical data conducted in Spanish, English, and Portuguese with their respective keywords (see Table 1), using the Boolean operators AND, OR, and quotation marks AND and OR with the symbol + and quotation marks for the search, as indicated in Table 2. In addition, the exploration of articles published between 2012 and 2022 was configured during April and August 2022 from the search in four databases, from which a total of 50,849 documents were obtained Web of Science (*n* = 566), Scopus (*n* = 102), EBSCO host (*n* = 33), and Redalyc (*n* = 31,733). To complement the selection process, a second search for articles updated until the end of 2022 was carried out in the Web of Science (4 articles), Scopus (1 article) and Redalyc (9 articles) databases. In total, 15 additional articles were identified that met the eligibility criteria.

**Table 1:** Keywords searched in the databases.

Spanish	English	Portuguese
Memoria colectiva	Collective memory	Memória coletiva
-	Memories/memory	Memória
Experiencia	Experience	Experiência
Cambio climático	Climate change	Alterações climáticas
Personas mayores	Elderly	Idoso

Made by the authors.

**Table 2:** Database search equations.

Search Equation	Search Articles 1	Search Articles 2
“memoria colectiva” AND “personas mayores” AND “cambio climático”	4	0
“memory” or “experience” AND “elderly” AND “climate change”	165	6
“memories” or “experience” AND “elderly” AND “climate change”	165	6
“memoria” OR “experiencia” AND “personas mayores” AND “cambio climático”	135	2
“memória” OR “experiência” AND “idoso” AND “alterações climáticas”	28	0
“collective memory” AND “elderly” AND “climate change”	10	0
“memória coletiva” AND “idoso” AND “alterações climáticas”	1	0
Total de artículos encontrados	508	14

Made by the authors.

### Article selection procedure

**Figure 1:**
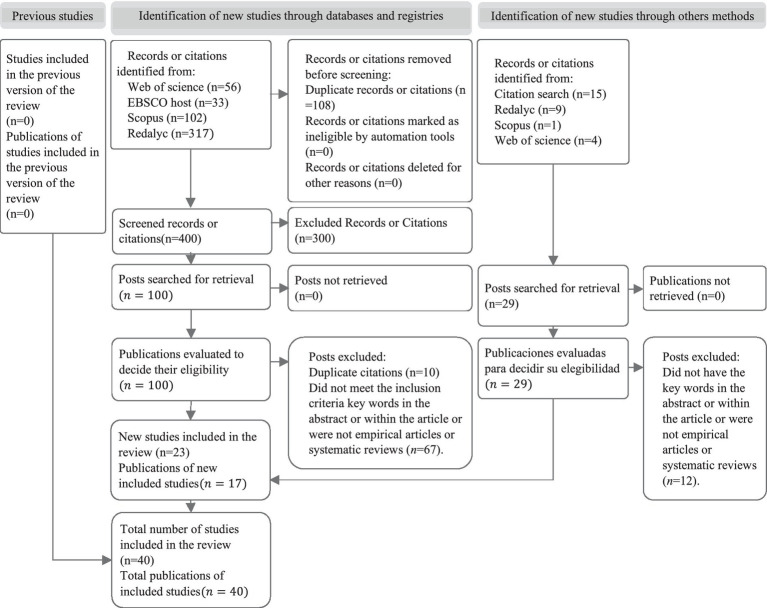
PRISMA flow diagram: literature identification and selection process. Prepared from Page et al. (25).

A selection was made in stages (see Figure 1). First, all the articles collected (*n* = 508) were compiled; second, the titles were read and duplicates were eliminated (*n* = 108); third, the titles, abstracts, keywords, and instruments used were read, eliminating those that did not meet the inclusion criteria (*n* = 300); fourth, a full-text reading was carried out, eliminating theoretical instrumental studies or those that did not focus their results on the collective experience and/or memory, climate change, and the older people (*n* = 67); fifth, a second search to obtain studies updated to 2022 from the databases and the search for citations (*n* = 29); and sixth, the last elimination was made of articles that did not meet the inclusion criteria (*n* = 12).

After the analysis and review of the selected articles (*n* = 40), an integrative synthesis of the selected works was carried out to compare the different studies, extracting: author/s and year of publication, country of the study, type of report or experience addressed, type of risk and/or disaster, methodology, sample, source of information, and whether it is primary or secondary.

## Results

Table 3 presents the synthesis of empirical studies, which are concentrated in Australia (13.2%), China (10.5%), the United States (7.9%), Mexico (7.9%), India (5.3%), and the United Kingdom (5.3%). In this context, Latin America and the Caribbean have gathered 18.9% of the studies, focusing their research on Mexico (7.9%), Chile (2.6%), and Ecuador (2.6%) while 10.5% of the remaining studies do not report the country of study.

**Table 3:** Synthesis of empirical studies.

Author/s	Study country	Type of memory or experience	Disasters or associated risks	Methodology	Sample	Source of information	Primary, secondary or mixed source?
Baldwin et al. (22)	Australia	Experience	Heat waves (urban heat islands)	Qualitative	Representatives of care centers for the elderly	Workshop/discussion group	Secondary
Iglesias Da Cunha et al. (17)	Spain	Lived experience, collective memory	High carbon emissions	Qualitative	Old people	Group interview	Primary
McNamara and Clissold (26)	Maldives	General and lived experience	droughts, tsunamis, earthquakes, floods, Tropical storms, saltwater Intrusion, changes in seasons, coastal erosion	Qualitative	Snowball sampling: 24 participants from Laamu Atoll and Malé	Semi-structured interview	Secondary
Chávez Alvarado and Sánchez González (27)	Mexico	Traumatic experience	Floods	Qualitative	Not probabilistic. 68 people aged 60 and over with disabilities who had suffered at least one flood, and residents of private homes in areas at risk of flooding	Interwiew	Primary
García-Valdez et al. (28)	Latin America and the Caribbean	Spatial and everyday experience	Climate change, heat waves and migration	Qualitative	Articles from journals indexed in Web of Science and Scopus	Articles from journals indexed in Web of Science and Scopus	Secondary
Sánchez González (29)	Latin America	Space experience	Environmental migration	Qualitative	-	-	Secondary
Gilchrist and Gearey (30)	United Kingdom	Lived and historical experience	Lack of water resources: Drought	Qualitative	22 people age 55 or older who are of retirement age or have chosen to retire	Semi-structured interview	Mixed
Huang et al. (31)	China	Experience	Floods	-	-	Survey	Primary
Astill and Miller (10)	Australia	General, lived and past traumatic experience	Cyclones	Qualitative	Older people who have lived in the region for at least 10 years	Semi-structured interview, focus group	Primary
Haq and Gutman (32)	Nordic arctic	Overall experience	-	Qualitative	Seniors, researchers, and a number of local individuals and professionals who provide services to promote northern culture and the well-being of seniors	Interwiew	Mixed
Begum (7)	-	Overall experience	Extreme phenomena	-	-	-	-
Sampson et al. (9)	United States	General and evacuation experience	Hot spells	Qualitative	Detroit (22 community members and 22 government or nonprofit leaders); New York (25 community members and 21 nonprofit or government leaders); Philadelphia (27 community members and 9 non-profit or government leaders); and Phoenix (22 community members and 25 government or nonprofit leaders).	Semi-structured interview, workshop	Mixed
Sampson et al. (33)	United States	General, traumatic and lived experience	Flood	Qualitative	Snowball sampling: Heads of household over 18 years of age	Entrevista semiestructurada	Primary
Seebauer and Winkler (15)	Austria	Overall experience	Flood	Qualitative	79 households living in the relocation zone	Semi-structured interview	Primary
Vasseur et al. (16)	Canada	General and storm experience	Flood	Qualitative	74 participants	Semi-structured interview	Mixed
Gutsa (34)	Zimbabwe	Overall experience	Dry weather	Qualitative	10 elderly women heads of household	Focus group, interview	Secondary
Malak (8)	Bangladesh	Overall experience	Cyclone	Qualitative	Older people and key stakeholders (local elected representatives, school teacher, NGO officials, local state executive, community health provider and social worker)	Semi-structured interview, focus group discussion and oral histories in three villages (Lebubunia, Gabura and Dumuria)	Mixed
Nobert and Pelling (35)	England	Everyday and temporary experience	Heat waves	Qualitative	30 independent older people (68–95 years) and carers in the Boroughs of Islington, Waltham Forest and London during and after the 2013 heatwave	Semi-structured interview, first hand observation and document analysis	Mixed
Reckien and Petkova (36)	United States	Previous, local and drought experience	Previous, local and drought experience	Qualitative	Randomly selected, representative of the adult population of New York City in terms of gender and age	Online Questionnaire Survey	Mixed
Rich et al. (37)	Australia	General, lived and aging experience	Drought	Qualitative	15 Australian women over the age of 70	Mail survey	Primary
Zhu and Sun (24)	China y Japan	General, previous and life experience	Earthquake	Qualitative	Elderly people over 75 years	Earthquake databases, earthquake shaking map and census	Primary
Stafford and Baldwin (38)	-	General and lived experience	-	Qualitative	96 peer-reviewed articles in English published between 2000 and 2016	Articles	-
Ogunbode et al. (39)	United Kingdom	Extreme weather experiences and staff	Extreme weather	Mixed	Specific oversampling of people in 5 flood-affected parts of the UK	Survey	Mixed
Yang et al. (14)	China	Personal experience	Air pollution	Qualitative	1,181 respondents from 3 cities in China	Survey	Primary
Loughnan et al. (40)	Australia	Personal experience	Heat waves	Mixed	Snowball sampling: Individuals 55 years or older, living independently, and able to speak and read English	Grupo focal, entrevista y registro de actividades diarias	Primary
Gray et al. (41)		Experience	-	Mixed	Silent generation1, baby boomers2, generation x3 and millennials4	Survey	Mixed
Brockie and Miller (11)	Australia	Life experience and previous	Flood	Qualitative	10 Brisbane seniors who were evacuated in the 2011 and 2013 floods	Interview	Primary
Rubio Aguilar (42)	Chile	General and life experience	Fire	Qualitative	Inclusion criteria: 1 affected elderly male living alone and 1 accompanied, 1 affected elderly female living alone and 1 accompanied	Interview	Primary
Gifford and Nilsson (43)	-	Childhood experience	Environmental damage	Qualitative	Research	Empirical articles	Primary
Yang et al. (44)	China	Emotional experience	Air pollution	-	University students who were with their grandparents	Affective Imaging, Self-Assessment Manikin (SAM), Beck Depression Inventory (DBI), State–Trait Anxiety Inventory (STAI), and Physiological Measurement	Secondary
Rojas Baltazar et al. (45)	México	Overall experience	Insuficiencia de suministros urbanos	Qualitative	-	Aplicación de escala	Primary
Torres Carral and Castillo López (46)	México	General and personal experience	Migration	Qualitative	16 people over 40 years of age	Semi-structured interview	Primary
Weitz et al. (47)	India	Overall experience	Heat waves	-	Elderly residents of an urban slum and elderly residents of rural villages in India (n = 130)	Interview and questionnaire	Primary
Chanza and Musakwa (48)	Zimbabwe	Lived and local experience	Storm, flood and drought	Qualitative	23 women and 14 men with an average age of 63 years	Semi-structured interview	Primary
Yang et al. (49)	China	Overall experience	Heat waves	-	Labor force with ages between 15 and 64 years	China Labor Force Dynamic Survey (CLDS)	Mixed
Sawangnate et al. (50)	Thailand	Past flood experiences	Flood	Mixed	Expert interview and community survey	Expert interview and community survey	Mixed
Crona et al. (51)	Fiji, Ecuador, New Zealand, Australia, United Kingdom and United States	Individual personal and historical experiences	Climate change	Mixed	Non-probability purposive sampling designed to capture only local residents (n = 29)	Location-Based Open Interview	Secondary
Ford et al. (52)	-	General and lived experience	Climate change	Qualitative	IPCC Articles	IPCC Articles	Secondary
Smith et al. (53)	India	General and crop experience	Decline in pollinators	Mixed	80 farmers who were trained and 50 farmers who were not trained	Group discussion	Mixed

“-’’: Not reported in studies. 1: People born between 1925 and 1945 (41). 2: People born between 1946 and 1964 (41). 3: People born between 1965 and 1981 (41). 4: People born between 1982 and 1999 (41). Made by the authors.

There was a greater development of research around the general experience of the older adult (34.3%), oriented to the knowledge acquired from a lifetime; the lived experience (14.9%), referring to what was lived around daily life at the time of the disaster; personal experience (6%), which is not influenced by third parties, but only the subjective attribution of the subject is conceived; previous experience (4.5%), understood as the information that was obtained before the event, either directly or indirectly; life experience (4.5%), understood as the knowledge generated from what has been learned, directly or through the story provided by other people; local experience (3%), alluding to the learning generated from the environment and from what was lived in the community of origin; collective memory (3%); daily experience (3%), referring to what was lived around the daily chores within the home during the disaster; spatial experience (3%), understood as knowledge based on the area inhabited and beyond the home of origin, for example, knowledge based on what has been experienced as an immigrant.

Although there are several disasters of natural origin associated with climate change, studies on the experience of the older adult have mainly addressed floods (18.8%), heat waves (10.4%), storms (6.3%), droughts (6.3%), and climate change, in general (6.3%).

In terms of methodology, there was a predominance of qualitative studies (67.5%) that used interviews (52.5%) as the main technique. In relation to the role of older persons in the research analyzed, three subgroups were identified: i) older persons as a primary source (41%), ii) older persons as a mixed source, in which other key agents who live or work with the older adult population were incorporated (30.8%), and iii) older persons as an indirect source, that is, only through key agents (25.6%).

In another area, research has reported various lessons learned from the experience and/or collective memory of older people after a disaster (see Table 4). Through experience and from the cognitive point of view, this age group has been valued as a historical source due to all the knowledge they have obtained from their experience of a disaster (15%) and a greater risk perception after experiencing a disaster (12.5%). However, the valence effect (12.5%) has also been highlighted, which causes a greater risk in the population, making them believe that they have a lower risk of experiencing a negative event compared to other people (36). At *an emotional level*, emotional resilience stands out (15%), understood as the ability to not be affected or to overcome more quickly the worry, uncertainty, and anxiety caused by a disaster (41) and the one that can recognize the traumatic event (12.5%). At the *social level*, it stands out that after a disaster, the older adult tend to form a group identity (10%), favoring the social support obtained (7.5%) and the manifest need for support (7.5%). In other words, social cohesion increases and, consequently, it would favor the generation of new support networks, whether intra- or extra-familial (28). At the *behavioral level*, behavioral adaptation is revealed through generalized coping strategies (25%), the change in gender roles (20%), and a greater general adaptation to climate change (20%). At the *spatial level*, a greater attachment to the place where the older adult live (15%) and an active role in planning (10%) were observed.

**Table 4:** Learning obtained through experience or collective memory after a disaster according to studies.

The way of knowledge	Category	Learning obtained	Frequency	Authors
Experience	Cognitive	Determinant of psychological well-being	4	García-Valdez et al. (28), Haq and Gutman (7), Malak et al. (8), Sánchez González (29)
		Spirituality Connection	4	Gifford and Nilsson (43), Malak et al. (8), Rubio Aguilar (42), Sánchez González (29)
		Use of common sense	1	Nobert and Pelling (35)
		Gambler fallacy1	4	Brockie and Miller (11), Reckien and Petkova (36), Rich et al. (37), Viglione et al. (19)
		Valence effect2	5	Brockie and Miller (11), Loughnan et al. (40), Petkova et al. (54), Reckien and Petkova (36), Sawangnate et al. (50), Yang et al. (49)
		Indigenous knowledge (mitigation/adaptation)	2	Ford et al. (52), Smith et al. (53)
		Global cultural competence	1	Crona et al. (51)
		Risk perception	5	Brockie and Miller (11), Crona et al. (51), Gifford and Nilsson (43), Huang et al. (31), Ogunbode et al. (39), Sampson et al. (33)
		Positive assessment of the past	1	Rubio Aguilar (42)
		Common sense of history	4	Brockie and Miller (11), Gray et al. (41), Sánchez González (29), Zhu and Sun (24)
		Older person as a historical source	6	Chanza and Musakwa (48), Gutsa (34), Sampson et al. (33), Sawangnate et al. (50), Smith et al. (53), Torres Carral and Castillo López (46)
		Resignify what has been lived	1	McNamara and Clissold (26)
	Emotional	Home meaning and satisfaction	1	García-Valdez et al. (28)
		Emotional resilience	6	Brockie and Miller (11), Rich et al. (37), Rubio Aguilar (42), Sampson et al. (33), Seebauer and Winkler (15), Zhu and Sun (24)
		Feeling of loss and instability	1	Rich et al. (37)
		Recognition of traumatic event	5	Brockie and Miller (11), Gray et al. (41), Huang et al. (31), Rich et al. (37), Seebauer and Winkler (15)
	Social	Determinant of social support obtained	3	García-Valdez et al. (28), Haq and Gutman (7), Malak et al. (8)
		Manifest need for support	3	Brockie and Miller (11), Sampson et al. (33), Sawangnate et al. (50)
		Loss of community networks	1	Rich et al. (37)
		Deterioration of family cohesion	1	Rich et al. (37)
		Social change due to environmental migration	1	Begum (32)
		Climate change communication	2	Sawangnate et al. (50), Yang et al. (14),
		Group identity	4	Iglesias Da Cunha et al. (17), McNamara and Clissold (26), Ogunbode et al. (39), Seebauer and Winkler (15)
		Greater group connection	2	Baldwin et al. (22), Brockie and Miller (11)
	Behavioral	Determinant of physical well-being	4	García-Valdez et al. (28), Haq and Gutman (7), Malak et al. (8), Sánchez González (29)
		Service provision	1	McNamara and Clissold (26)
		Pro-environmental behavior (mitigation)	5	Chanza and Musakwa (48), Crona et al. (51), Gifford and Nilsson (43), Gray et al. (41), Smith et al. (53)
		Follow recommendations from authorities	3	Chávez Alvarado and Sánchez González (27), Nobert and Pelling (35), Sawangnate et al. (50)
		Digital literacy	1	Sawangnate et al. (50)
		Conscious volunteering	1	Gilchrist and Gearey (30)
		Generation of changes according to assets	1	Malak et al. (8)
		Coping strategies (adaptation in general)	10	Brockie and Miller (11), García-Valdez et al. (28), Huang et al. (31), Malak et al. (8), Rubio Aguilar (42), Sampson et al. (9), Sampson et al. (33), Sawangnate et al. (50), Stafford & Baldwin (38), Zhu and Sun (24)
		Double presence of women	8	Begum (32), Chanza and Musakwa (48), Crona et al. (51), Gutsa (34), Malak et al. (8), Rich et al. (37), Sánchez González (29), Vasseur et al. (16), Weitz et al. (47)
		Promote necessary changes at a general level (psychological adaptation)	8	Begum (32), García-Valdez et al. (28), Huang et al. (31), Iglesias Da Cunha et al. (17), Nobert and Pelling (35), Smith et al. (53), Stafford and Baldwin (38), Sánchez González (29)
	Space	Environment optimization	2	Haq and Gutman (7), Sánchez González (29)
		Attachment to place	6	Brockie and Miller (11), García-Valdez et al. (28), Gifford and Nilsson (43), Rubio Aguilar (42), Seebauer and Winkler (15), Sánchez González (29)
		Active role in planning	4	Rojas Baltazar et al. (45), Smith et al. (53), Sánchez González (29), Zhu and Sun (24)
		Aging in place	3	Ford et al. (52), García-Valdez et al. (28), Huang et al. (31)
		Belonging to a symbolic space that no longer exists	3	Brockie and Miller (11), Ford et al. (52), Sánchez González (29)
		Green infrastructure as a mitigator	1	Baldwin et al. (22)
Collective memory	General	Hope for a better future	1	Iglesias Da Cunha et al. (17)
		Promote joint actions	1	Iglesias Da Cunha et al. (17)

^*^The definition of apprenticeships can be reviewed in depth in the attached section. 1: Cognitive bias that causes a person to consider that they are at less risk of experiencing a negative event compared to other groups (36). 2: Belief that if an event occurs more frequently over time, it will happen less frequently in the future (36). Made by the authors.

Regarding collective memory, it was identified that, after a disaster, older people gained greater hope for the future, seeing it as a better future, and from this, they began to promote actions together with their peers to overcome the circumstances of risk of natural origin. However, this learning involved only 2.5% of recent research. Finally, regarding the characteristics of the other learning obtained, see Supplementary material S1 for their definitions.

## Discussion

Systematized scientific evidence shows that it has recently begun to be understood that the physical and emotional wellbeing of the older adult can be influenced by controlling the environment (34). However, it is paradoxical that this manifestation, on many occasions, does not come directly from this age group, but from key agents who interact with the older adult; it would therefore be interesting to know the perspective of the protagonists themselves. Therefore, it is essential that in future studies, the knowledge provided by the older adult can be privileged, recognizing the importance of their life histories and favoring the active role in their development and wellbeing (28).

It has been observed that when people can tell their stories of trauma, they can recover and resignify what happened more easily, emphasizing the confidence they have achieved in their strength and in the ability to manage the resources they were able to deploy in the face of a certain disaster, thus adapting to the post-event physical and social environment, enhancing perceived empowerment (28). In connection with the above, after experiencing a disaster, it would be beneficial for this group to have listening spaces, even more so given the perception of loneliness that has been manifested in various studies (11, 41, 55).

On the other hand, there is a gap in the literature regarding the study of the previous experiences and collective memory of the older adult in the face of risks and/or disasters of natural origin, mainly those caused by climate change, reflected in the low number of empirical studies found. In this way, it is important to delve into this issue, and, through it, enhance the agency capacity of the older adult, especially in those places where they are at greater risk of disaster (45).

When making a comparison between the number of studies that address experience and collective memory, the difference observed is significant, since when looking for an explanation, some studies express their preference for investigating collective memory only when it refers to phenomena that have greater social and psychological significance for the community (56, 57), prioritizing those events that are considered more “collectively representative” among the population. Therefore, some of the “silent risks” of climate change (such as heat waves and frosts) do not have great research relevance so far (58), increasing the scientific debt toward the older adult population. However, it is important to highlight that environmental gerontology, a relatively new discipline (especially in Latin America), has been focusing on carrying out multidisciplinary work that addresses this debt through the understanding, analysis, and optimization of the relationship between the physical-social environment and the aging person (59, 60). In this way, it is intended to raise awareness about the phenomenon of aging and the importance of building friendly environments that reinforce support networks within the community (29, 61).

In another area, it is possible to point out that much of the literature reviewed in the field of environmental gerontology and older people have been built in developed countries, evidencing a scarcity of research focused on the population of Latin America and the Caribbean (58, 62). In the same way, these studies are developed under qualitative methodologies, leaving aside other research perspectives (quantitative or mixed designs, for example), so it is necessary to expand the research development from this perspective (6), generating new knowledge based on the permanent change of the physical and social environments, even more so when they are in danger of experiencing a disaster in the short, medium or long term (59).

In short, it is essential to obtain adequate knowledge so that this same age group can generate the necessary strategies to adapt and protect itself from climate risks (13), minimizing susceptibilities by strengthening its capacity for the agency (28).

## Conclusion

Previous disaster experiences and collective memory have been identified as adaptive capacities in older people (63), which has been expressed through the learnings obtained after some potentially traumatic event of natural origin. Specifically, negatively valenced emotions, be it fear and anger, and perceived self-efficacy would drive precautionary attitudes and behaviors (8, 53). In this case, the deployment of coping skills would be motivated by the level of involvement of the person in the face of the event experienced, which would amplify the perception of risk and the organization of their resources to deal functionally with climate change (43). Similarly, Sandoval-Obando (64) describes generative coping as that set of actions and tasks deployed by the older adult in the face of potentially traumatic events (pandemic for example), in which solidarity, trust, social participation, reciprocity, and mutual support give them a greater degree of self-efficacy and social support in the face of these events (65).

The agency capacity of the older adult in the face of disasters of natural origin, either individually or collectively, favors adaptation processes through experience and the respective personal meaning of what they have experienced (24, 31), actively empowering itself during the aging process. In other words, on a personal level, the older adult can value and make decisions about their lives and know how to act in the face of danger, beyond their family, and, on a social level, allows them to be part of the community, integrating and actively participating in their environment (66–68). In short, the empowerment of the older adult makes it possible to overcome ageism conceptions of old age, reducing vulnerability indices to the risks generated by climate change, and at the same time, allows them to be recognized as an age group of enormous historical-cultural value for future generations (30, 48).

By way of reflection, it is possible to point out that the experience and collective memory of this age group in the face of potentially traumatic events of natural origin, emerges as a resilient post-disaster attitude, thanks to the positive assessment they establish with themselves, in addition to the recognition and appreciation of their knowledge and personal resources (12, 42), becoming a determinant of individual/social resilience (69). At the same time, it would favor a better psychological adjustment and less emotional distress after a disaster (11). Finally, the experience of aging in changing environments as a consequence of climate change can stimulate the emergence of functional behaviors and challenging tasks for the older adult, contributing to their adaptive process (59).

## Data availability statement

The original contributions presented in the study are included in the article/Supplementary material, further inquiries can be directed to the corresponding author.

## Author contributions

CN-V and JS-D contributed to conception and design of the study. CN-V organized the database. CN-V, JS-D, and ES-O wrote sections of the manuscript. All authors contributed to manuscript revision, read, and approved the submitted version.

## Funding

This work was financed by the National Agency for Research and Development (ANID)/FONDECYT of Initiation No. 11200683 and the UBB2095 project “Strengthening capacities and the role of collective memories in the face of disaster risk processes of the elderly” of the Universidad del Bío Bío.

## Conflict of interest

The authors declare that the research was conducted in the absence of any commercial or financial relationships that could be construed as a potential conflict of interest.

## Publisher’s note

All claims expressed in this article are solely those of the authors and do not necessarily represent those of their affiliated organizations, or those of the publisher, the editors and the reviewers. Any product that may be evaluated in this article, or claim that may be made by its manufacturer, is not guaranteed or endorsed by the publisher.
