# Supplementary material for: Experience and local memory of older people in the face of disasters: a systematic review
Source: Front Public Health. 2023 May 24;11:1163561. doi: 10.3389/fpubh.2023.1163561 (PMC10246736; doi:10.3389/fpubh.2023.1163561)
Supplement: Supplementary file 1 [file Data_Sheet_1.docx]

**Annex**

**A**

**Active role in planning:** Proactive and committed participation of a person or group in the development and implementation of a plan or project oriented from and towards older people (Baldwin et al., 2020).

**Aging in place:** circumstances that determine identity and attachment to place (Sánchez-González, 2009). In this regard, knowledge has accumulated regarding environmental identity in aging, the meaning of place, and spatial experiences associated with changes in one's environment or due to relocation (Astill & Miller, 2018), whereby older people accommodate, adjust and relocate the pressures of the physical-social environment according to their capabilities in order to achieve adaptation and aging where they wish (García-Valdez et al., 2019).

**B**

**Belonging to a symbolic space that no longer exists:** Feeling part of an environment, space or place that he or she used to inhabit or recreate in that no longer exists because of the disaster, which place that was important and made him or her feel part of a group or community (Kemen et al., 2021).

**C**

**Climate change communication:** Process of informing, educating and raising awareness about the impacts of climate change and the measures that can be taken to reduce its impact, in this way, people manage to interpret and recognize climate change towards their peers, identifying risks and prevention measures (Arias Ortega & Rosales Romero, 2019).

**Community networks:** Shaped by the participation of older people in activities that favor social cohesion (Chávez-Alvarado & Sánchez-González, 2016), and the supportive links between people with shared identities (Wolf et al., 2010).

**Connection with spirituality:** Beliefs and practices of religiosity of people in the face of disaster or trauma situations, performing actions to lessen distress or have comfort (Lomas et al., 2015).

**Conscious volunteering:** Directed act or performance of agency to provide support in times of crisis and responds directly to the recognition that some government institutions cannot or fail to take charge of what is happening (Gilchrist & Gearey, 2019).

**Coping strategies:** actions taken to face and cope with adverse situations that may occur during or after a catastrophic event. These strategies may include planning ahead, seeking social support, remaining calm, and adapting to the new circumstances, becoming a stabilizing factor in response to and during the crisis (Sandoval-Díaz et al., 2022).

**D**

**Determinant of social support obtained:** This refers to the quantity and quality of emotional, tangible and informational support that a person receives from his or her environment during and after a disaster. This support can come from friends, family, neighbors, community organizations, government agencies, among others (Navarrete Valladares & Sandoval-Díaz, 2022; Sampson et al., 2013).

**Determinant of physical well-being:** State mediated by the health of the person and their ability to recover and maintain their physical health after a disaster, being hindered by any factor that may place the person at risk (Burns & Machado Des Johansson, 2017).

**Determinant of psychological well-being:** subjective state mediated by the person's ability to recover and recover emotionally, which can be interfered with by the severity and exposure of the disaster (Hobfoll et al., 2007).

**Digital literacy:** Refers to a person's ability to use digital technologies effectively and responsibly (Sunkel et al., 2012). It includes an understanding of how computers, the internet, and digital tools work. In the context of disaster risk, digital literacy plays an important role in the exchange and dissemination of information through the use of technologies (Sawangnate et al., 2022).

**Driving joint actions:** Collaboration and coordination between different entities, such as governmental, non-governmental and civil society organizations, to prepare for and respond effectively to emergency situations or natural disasters (Asian Disaster Reduction Center, 2015).

**E**

**Emotional resilience:** A person's ability to adapt and overcome adverse or stressful situations, maintaining a balanced and constructive emotional state (Sierra, 2016).

**Environmental optimization:** Implementation of planning and design measures that seek to reduce the vulnerability of communities and increase their resilience to extreme events, such as disasters (Tidball & Krasny, 2013).

**F**

**Family cohesion:** Strength and quality of sentimental and emotional ties between family relationships (Quintero Velásquez, 2005).

**Follow recommendations of authorities:** Aimed at complying with and applying suggestions, guidelines or instructions issued by persons or entities with authority in order to maintain the safety, health and well-being of the population or group they are addressing during or after a disaster risk (Tilstra et al., 2021).

**G**

**Gambler's fallacy:** Cognitive bias that causes a person to believe that he or she is at less risk of experiencing a negative event compared to other groups or individuals (Reckien & Petkova, 2019).

**Generation of changes according to assets:** Management of the change process in different areas of personal life after living a traumatic experience or a catastrophic event (Chávez-Alvarado & Sáchez-González, 2016). In addition, it involves the identification of damage due to the disaster, the assessment of options and resources available for recovery and reconstruction, decision making and implementation of actions to achieve physical, emotional and psychological recovery (Sierra, 2016).

**Global cultural competence:** The ability of members of a culture to know about cultural events beyond their own context (UNESCO, 2013). In addition, it can determine whether observed variability in an individual's beliefs is of cultural origin or whether it only reflects differences in individual familiarity with elements of their own culture (Crona et al., 2013).

**Green infrastructure as a mitigator:** Urban planning and design approach that incorporates natural elements to provide environmental and social benefits to reduce the impact of climate change and improve the resilience of cities in terms of temperature and intensity of extreme events (Rojas Baltazar et al., 2022).

**Group identity:** Feeling of being part of a community or group, and the influence of this is much stronger on people's behaviors and beliefs than scientific competence in relation to climate change (Iglesias Da Cunha et al., 2020).

**Group connection:** referring to how individuals, families, groups, communities respond to and manage change among themselves and with respect to the ecosystems and environments with which they are connected (Gilchrist & Gearey, 2019).

**H**

**Hope for a better future:** positive attitude or belief that whatever happens in the future will improve, however, it may lead to a minimization of the severity of the situation and a lack of preparedness to face the long-term consequences of climate change (Li & Monroe, 2019).

**I**

**Indigenous knowledge:** From ethnoecology, it is understood as the belief system, set of knowledge and productive practices of a community or indigenous person with the objective of adapting to climate change (Torres Carral & Castillo López, 2022). In turn, indigenous peoples, through local experience, are able to maintain a distinct, systematic, localized and place-based environmental knowledge over long periods of time (Chanza & Musakwa, 2022).

**M**

**Manifest need for support:** Series of structural and functional aspects derived from the maintenance of social relationships, and even by the direct attempt to manage and reduce the consequences on health of a stressful event such as socio-natural disasters (Navarrete Valladares & Sandoval-Díaz, 2022), therefore, the purpose of social support is mediated by ensuring that the appropriate conditions are propitiated towards an improvement in the quality of life of the individual (Aranda & Moreno, 2013).

**Meaning and satisfaction of home:** Attachment to place and state of well-being in the place that the older person inhabits in his or her daily life (Seebauer & Winkler, 2020).

**O**

**Older person as historical source:** conception of the older person as a person with a lot of experience, which gives them advantages in their knowledge of events that have occurred previously (Brockie & Miller, 2017).

**P**

**Place attachment:** emotional attachment to an area where people prefer to stay and where they feel comfortable and safe (Seebauer & Winkler, 2020); Sandoval-Díaz et al., 2022a).

**Positive appraisal of the past:** Similar to re-signifying what has been experienced, but the difference lies in the fact that there is no attempt to re-interpret what has been experienced, but rather to recall the past and value more what was experienced before than at present (Seebauer & Winkler, 2020).

**Promote necessary changes at a general level:** To undertake on one's own initiative and encourage changes in behavior or the environment to adapt to disaster risks (Regional Observatory for Development Planning in Latin America and the Caribbean, 2020).

**Pro-environmental behavior:** Set of actions aimed at conserving and protecting the integrity of environmental resources with the objective of minimizing negative impacts (Martínez-Soto, 2006).

**R**

**Recognition of traumatic event:** Process of identifying and validating a person's (and one's own) experience that has been exposed to a traumatic event and that this has generated a great emotional and psychological impact (Astill & Miller, 2018).

**Resignifying the lived:** Reinterpreting traumatic experiences that have been lived during or after a disaster. This process involves finding a more positive meaning to what happened, identifying lessons learned, and using the experience to grow and become emotionally stronger (United Nations, 2022).

**Risk perception:** The way in which people perceive and evaluate the probability and consequences of an extreme event. This perception is influenced by factors such as previous experience, knowledge, culture, trust in authorities and the media, among others (Seebauer & Winkler, 2020).

**S**

**Sense of common history:** Sense that is formed due to sharing different experiences together with a group, which makes those who participated in a similar experience feel understood and empathetic with the suffering or experience of the other (Malak et al., 2020).

**Sense of loss and instability:** Post-disaster sensation that generates anxiety and distress in the older person, often caused by the loss of the material goods they once owned (Weitz et al., 2022).

**Service provision:** Refers to the delivery of a set of activities or processes that meet the needs or demands of clients or users. These services may be offered by a company, organization or government entity (Baldwin et al., 2020).

**Social change due to environmental migration:** Transformations that occur in communities and societies as a result of the forced migration of people due to natural disasters or environmental impacts (Black et al., 2013).

**U**

**Use of common sense:** Its purpose is to provide a socially shared meaning to the world, facilitating communication and cultivating identity within a social group (Iglesias Da Cunha et al., 2020), in turn, it allows using the knowledge of the past to give shape and recognition to the present (Sampson et al., 2013).

**V**

**Valence effect:** Psychological phenomenon that forms the belief that, if a disaster occurs more frequently during a certain time, it will happen less frequently in the future (Reckien & Petkova, 2019).

**W**

**Women's double presence:** Situation in which women face additional challenges and have multiple and overlapping roles during and after a disaster, and, in addition to having to deal with the consequences of risk, women also face specific challenges related to their gender and discrimination in the distribution of resources and relief services (García & López, 2013; UN Women, 2013).

**References**

Arias Ortega, M. Á., & Rosales Romero, S. (2019). Educación ambiental y comunicación del cambio climático. Una perspectiva desde el análisis del discurso. *Revista mexicana de investigación educativa*, *24*(80), 247-269.

Asian Disaster Reduction Center. (2015). Sendai framework for disaster risk reduction 2015–2030. *United Nations Office for Disaster Risk Reduction: Geneva, Switzerland*.

Astill, S., & Miller, E. (2018). 'The trauma of the cyclone has changed us forever': Self-reliance, vulnerability and resilience among older Australians in cyclone-prone areas [Article]. *Ageing and Society*, *38*(2), 403-429. <https://doi.org/10.1017/S0144686X1600115X>

Baldwin, C., Matthews, T., & Byrne, J. (2020). Planning for Older People in a Rapidly Warming and Ageing World: The Role of Urban Greening [Article]. *Urban Policy & Research*, *38*(3), 199-212. <https://doi.org/10.1080/08111146.2020.1780424>

Black, R., Arnell, N. W., Adger, W. N., Thomas, D., & Geddes, A. (2013). Migration, immobility and displacement outcomes following extreme events. *Environmental Science & Policy*, *27*, S32-S43.

Brockie, L., & Miller, E. (2017). Understanding older adults’ resilience during the Brisbane floods: social capital, life experience, and optimism. *Disaster medicine and public health preparedness*, *11*(1), 72-79.

Burns, T. R., & Machado Des Johansson, N. (2017). Disaster risk reduction and climate change adaptation—A sustainable development systems perspective. *Sustainability*, *9*(2), 293.

Chanza, N., & Musakwa, W. (2022). Indigenous local observations and experiences can give useful indicators of climate change in data-deficient regions [Article]. *Journal of Environmental Studies and Sciences*. <https://doi.org/10.1007/s13412-022-00757-x>

Chávez-Alvarado, R., & Sáchez-González, D. (2016). Envejecimiento vulnerable en hogares inundables y su adaptación al cambio climático en ciudades de América Latina: el caso de Monterrey. *Papeles de Población*, *22*(90), 9-42. <https://www.redalyc.org/articulo.oa?id=11249884002> (IN FILE)

Crona, B., Wutich, A., Brewis, A., & Gartin, M. (2013). Perceptions of climate change: Linking local and global perceptions through a cultural knowledge approach. *Climatic change*, *119*(2), 519-531.

García-Valdez, M. T., Román-Pérez, R., & Sánchez-González, D. (2019). Envejecimiento y estrategias de adaptación a los entornos urbanos desde la gerontología ambiental. *Estudios Demográficos y Urbanos*, *34*(1), 101-128. <https://www.redalyc.org/articulo.oa?id=31258446005> (IN FILE)

García, E. M., & López, E. R. (2013). Mujeres, Desastres y Cambio Climático. In: Vínculos y Desafíos. CEPAL.

Gilchrist, P., & Gearey, M. (2019). Reframing rural governance: gerontocratic expressions of socio-ecological resilience. *Ager. Revista de Estudios sobre Despoblación y Desarrollo Rural*(27), 103-127. <https://www.redalyc.org/articulo.oa?id=29662605008> (IN FILE)

Hobfoll, S. E., Watson, P., Bell, C. C., Bryant, R. A., Brymer, M. J., Friedman, M. J., Friedman, M., Gersons, B. P. R., De Jong, J. T. V. M., & Layne, C. M. (2007). Five essential elements of immediate and mid–term mass trauma intervention: Empirical evidence. *Psychiatry*, *70*(4), 283-315.

Iglesias Da Cunha, L., Pardellas Santiago, M., & GradaÍLle Pernas, R. (2020). Públicos invisibles, espacios educativos improbables: el proyecto "Descarboniza! que non é pouco..." como educación para el cambio climático [Article]. *Invisible Audiences, Unlikely Educational Spaces: The "Descarboniza! que non é pouco..." projects as education for climate change*(36), 81-93. <https://doi.org/10.7179/PSRI_2020.36.05>

Kemen, J., Schäffer-Gemein, S., Grünewald, J., & Kistemann, T. (2021). Heat Perception and Coping Strategies: A Structured Interview-Based Study of Elderly People in Cologne, Germany. *International journal of environmental research and public health*, *18*(14), 7495.

Li, C. J., & Monroe, M. C. (2019). Exploring the essential psychological factors in fostering hope concerning climate change. *Environmental Education Research*, *25*(6), 936-954.

Lomas, T., Cartwright, T., Edginton, T., & Ridge, D. (2015). A qualitative analysis of experiential challenges associated with meditation practice. *Mindfulness*, *6*, 848-860.

Malak, M. A., Sajib, A. M., Quader, M. A., & Anjum, H. (2020). "We are feeling older than our age": Vulnerability and adaptive strategies of aging people to cyclones in coastal Bangladesh. *International Journal of Disaster Risk Reduction*, *48*, Article 101595. <https://doi.org/10.1016/j.ijdrr.2020.101595>

Martínez-Soto, J. (2006). *Comportamiento proambiental. Una aproximación al estudio del desarrollo sustentable con énfasis en el comportamiento persona-ambiente*. Red Theomai.

Navarrete Valladares, C., & Sandoval-Díaz, J. (2022). El rol del apoyo social frente al cambio climático en la población mayor. *Revista Pensamiento y Acción Interdisciplinaria*, *8*(2), 13-33. <https://doi.org/10.29035/pai.8.2.13>

Observatorio Regional de Planificación para el Desarrollo de América Latina y el Caribe. (2020). La planificación para el desarrollo y la gestión del riesgo de desastres. In *Notas de planificación para el desarrollo*: CEPAL.

Organización de las Naciones Unidas. (2022). *La resiliencia de las personas mayores en un mundo cambiante*. Organización de las Naciones Unidas. Retrieved 15 de noviembre from <https://www.un.org/es/observances/older-persons-day>

Quintero Velásquez, A. M. (2005). Resiliencia: Contexto no clínico para trabajo social. *Revista Latinoamericana de Ciencias Sociales, Niñez y Juventud*, *3*(1), 73-94.

Reckien, D., & Petkova, E. P. (2019). Who is responsible for climate change adaptation? *Environmental Research Letters*, *14*(1), Article 014010. <https://doi.org/10.1088/1748-9326/aaf07a>

Rojas Baltazar, A., Chung Alonso, P., & Correa Fuentes, D. A. (2022). Servicios urbanos para la construcción de resiliencia en los espacios públicos de tipo abierto en México. *Vivienda y Comunidades Sustentables*(11), 23-49. <https://www.redalyc.org/articulo.oa?id=665170661002> (IN FILE)

Sampson, N. R., Gronlund, C. J., Buxton, M. A., Catalano, L., White-Newsome, J. L., Conlon, K. C., O'Neill, M. S., McCormick, S., & Parker, E. A. (2013). Staying cool in a changing climate: Reaching vulnerable populations during heat events [Article]. *Global Environmental Change*, *23*(2), 475-484. <https://doi.org/10.1016/j.gloenvcha.2012.12.011>

Sánchez-González, D. (2009). Contexto ambiental y experiencia espacial de envejecer en el lugar: el caso de Granada. *Papeles de población*, *15*(60), 175-213.

Sandoval-Díaz, J., Monsalves, S., & Vejar, V. (2022). Capacidades y capital social ante un riesgo natural en personas mayores: el caso del Complejo Volcánico Nevados de Chillán, Chile. *Perspectiva Geográfica*, *27*(2).

Sawangnate, C., Chaisri, B., & Kittipongvises, S. (2022). Flood Hazard Mapping and Flood Preparedness Literacy of the Elderly Population Residing in Bangkok, Thailand. *WATER*, *14*(8), Article 1268. <https://doi.org/10.3390/w14081268>

Seebauer, S., & Winkler, C. (2020). Should I stay or should I go? Factors in household decisions for or against relocation from a flood risk area [Article]. *Global Environmental Change*, *60*, Article 102018. <https://doi.org/10.1016/j.gloenvcha.2019.102018>

Sierra, M. T. C. (2016). Resiliencia, bienestar y aprendizaje a lo largo de la vida. *Revista INFAD de Psicología. International Journal of Developmental and Educational Psychology.*, *1*(2), 161-170.

Sunkel, G., Trucco, D., & Cepal, N. U. (2012). Las tecnologías digitales frente a los desafíos de una educación inclusiva en América Latina: Algunos casos de buenas prácticas.

Tidball, K. G., & Krasny, M. E. (2013). *Greening in the red zone: disaster, resilience and community greening*. Springer Science & Business Media.

Tilstra, M. H., Tiwari, I., Niwa, L., Campbell, S., Nielsen, C. C., Allyson Jones, C., Vargas, A. O., Bulut, O., Quemerais, B., Salma, J., Whitfield, K., & Yamamoto, S. S. (2021). Risk and resilience: how is the health of older adults and immigrant people living in canada impacted by climate-and air pollution-related exposures? [Review]. *International Journal of Environmental Research and Public Health*, *18*(20), Article 10575. <https://doi.org/10.3390/ijerph182010575>

Torres Carral, G. A., & Castillo López, S. (2022). Milpa y saberes mayas en San Sebastián Yaxché, Peto, Yucatán. *Estudios de Cultura Maya*, *LIX*, 171-189. <https://www.redalyc.org/articulo.oa?id=281371269006> (IN FILE)

UN Women. (2013). Brief on Women, Girls and Disasters. In: United Nations Entity for Gender Equality and the Empowerment of Women.

UNESCO. (2013). Intercultural competences: A conceptual and operational framework. In: UNESCO.

Weitz, C. A., Mukhopadhyay, B., & Das, K. (2022). Individually experienced heat stress among elderly residents of an urban slum and rural village in India. *INTERNATIONAL JOURNAL OF BIOMETEOROLOGY*, *66*(6), 1145-1162. <https://doi.org/10.1007/s00484-022-02264-8>
